# Congenital Hypothyroidism Caused by a PAX8 Gene Mutation Manifested as Sodium/Iodide Symporter Gene Defect

**DOI:** 10.4061/2010/619013

**Published:** 2009-12-09

**Authors:** Wakako Jo, Katsura Ishizu, Kenji Fujieda, Toshihiro Tajima

**Affiliations:** ^1^Department of Pediatrics, Hokkaido University School of Medicine, N15, W7, Sapporo, Hokkaido 060-8638, Japan; ^2^Department of Pediatrics, School of Medicine, Asahikawa Medical College, 2-1-1-1 Midorigaoka Higashi, Asahikawa, Hokkaido 078-8510, Japan

## Abstract

Loss-of-function mutations of the PAX8 gene are considered to mainly cause congenital hypothyroidism (CH) due to thyroid hypoplasia. However, some patients with PAX8 mutation have demonstrated a normal-sized thyroid gland. Here we report a CH patient caused by a PAX8 mutation, which manifested as iodide transport defect (ITD). Hypothyroidism was detected by neonatal screening and L-thyroxine replacement was started immediately. Although ^123^I scintigraphy at 5 years of age showed that the thyroid gland was in the normal position and of small size, his iodide trapping was low. The ratio of the saliva/plasma radioactive iodide was low. He did not have goiter; however laboratory findings suggested that he had partial ITD. Gene analyses showed that the sodium/iodide symporter (NIS) gene was normal; instead, a mutation in the PAX8 gene causing R31H substitution was identified. The present report demonstrates that individuals with defective PAX8 can have partial ITD, and thus genetic analysis is useful for differential diagnosis.

## 1. Introduction

 Congenital hypothyroidism (CH) is the most common congenital endocrine disorder and occurs at rate of 1 in 3000–4000 births [1]. The causes of CH can be classified into two groups: thyroid developmental defects (thyroid dysgenesis) and inborn errors of thyroid hormone biosynthesis (dyshormonogenesis). Several genes responsible for thyroid dysgenesis have been identified such as TSH*β*-subunit, TSH receptor, the Gs *α*-subunit, TTF-1, TTF-2, GLIS3, and PAX8 [2]. Among them, PAX8 is a paired domain transcription factor and is expressed in the developing thyroid, kidney, and several areas of the central nervous system [3]. In addition to its role in normal thyroid development, PAX8 regulates the expression of genes encoding thyroglobulin (TG), thyroid peroxidase (TPO), and the sodium-iodide symporter (NIS) by binding to their promoter regions through its 128-amino acid paired domain [4, 5]. To date, several mutations of the PAX8 gene have been identified in CH patients [6–12]. Most of these mutations have caused thyroid dysgenesis; however, some patients with PAX8 mutation have a normal-sized thyroid gland [9, 10].

Iodide transport defect (ITD) is a rare disorder characterized by an inability of the thyroid to maintain a concentration difference of readily exchangeable iodide between the plasma and the thyroid. Diagnostic criteria for ITD include a variable degree of CH and goiter, low or absent radioiodide uptake, as determined by thyroid scintigraphy, and low iodide saliva to plasma (S/P) ratio [13, 14]. This disease is caused by mutations of the NIS gene [13–17]. 

 Here we report that a patient with a PAX8 mutation showed low iodide S/P ratio. The PAX8 mutation in this patient manifested as CH due to ITD.

## 2. Case Report

A male infant was born after full-term gestation by normal vaginal delivery from nonconsanguineous parents. His birth weight was 3342 g. The family history revealed no thyroid disease. There were no abnormal physical findings; however, neonatal mass screening using filter paper for congenital hypothyroidism at the age of 6 days showed a high level of thyroid stimulating hormone (TSH) (62.8 mU/L, normal <10 mU/L). The patient was referred to our hospital at the age of 17 days for further evaluation. At that time his body weight was 3950 g. Physical examination did not show any abnormal findings including goiter. Biochemical evaluation revealed that the serum TSH level was 202.7 mU/L, thyroxine, 91.3 nmol/L, and triiodothyronine, 1.88 nmol/L (Table 1). He was treated with levothyroxine (L-T4) at that time. At the age of 5 years, he underwent ^123^I scintigraphy after the discontinuing L-T4 treatment for one month. Although ^ 123^I scintigraphy showed a normally located thyroid gland, his 1-, 3-, and 24-hour ^123^I uptake values were 4.8%, 5.8%, and 2.9%, respectively (normal range, 10%–30%). ^123^I S/P ratios at 2 and 4 hours were 4.5 and 3.8, respectively (normal >20) (Table 1). He did not have goiter; however, his diagnosis was considered to be partial ITD based on low thyroidal iodide uptake and low S/P ratio. The patient is currently 23 years old, and he has never developed goiter during follow-up.

## 3. Method

After obtaining written consent from the patient and the patient's parent, genomic DNA was extracted from peripheral blood lymphocytes. The NIS and PAX8 genes were amplified by polymerase chain reaction (PCR) according to previously-described methods [6, 17]. After PCR amplification, the amplified products were subjected to direct sequencing.

## 4. Results

**Figure 1:**
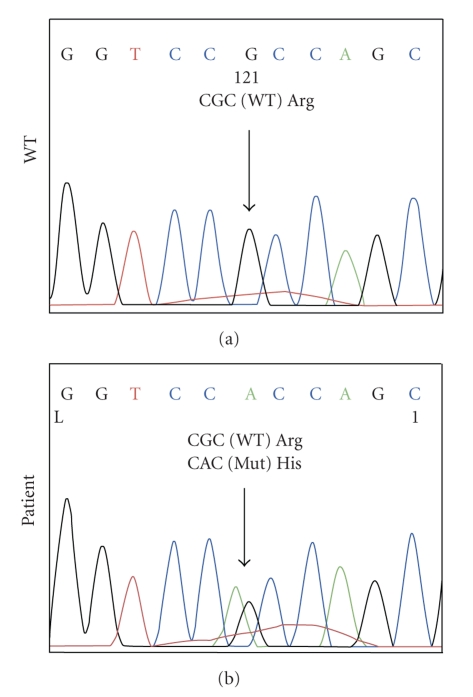
Sequence analysis demonstrated a C to A transition in the patient. This change substitutes histidine for arginine at residue 31 in the paired domain denoted by the arrow. WT: wild-type.

Analysis of the NIS gene revealed no nucleotide changes in the coding region nor in the exon-intron boundaries. Upon analysis of the PAX8 gene, we identified the patient washeterozygous for an arginine (CGC)-to-histidine (CAC) substitution at codon 31 (R31H), which was previously reported in a patient with CH [6] (Figure 1).

## 5. Discussion

In the present study, we reported a Japanese patient with R31H substitution in the PAX8 protein. Since the arginine at residue 31 is located in the paired domain in the PAX8 protein and is conserved among species, this amino acid substitution is thought to impair its DNA-binding activity, resulting in loss of function. It is of interest that a mutation at this residue was previously found in one Italian patient and one Japanese patient [6, 7]. These two patients were diagnosed as having thyroid hypoplasia as determined by ultrasonographic examination. We also identified R31H in another Japanese CH patient who had hypoplastic thyroid (unpublished data). To determine whether or not substitution of this arginine is frequent among Japanese patients with CH, further analysis is required. 

Defective mutations of the PAX8 gene have been considered to mainly cause thyroid hypoplasia. However, clinical heterogeneity has been observed among patients and even in the same family [9, 10]. One patient who had S54G substitution in of the PAX8 protein had organification defect and a normal-sized thyroid [10]. Since PAX8 plays a critical role in TPO expression during thyroid development [2, 4], impaired TPO gene expression due to PAX8 dysfunction may have led to organification defect in that patient. In this context, it may be possible that NIS expression is affected by mutations of the PAX8 gene, because a PAX8 binding site was found in the far-upstream enhancer region of the human NIS gene and PAX8 was required for activation of NIS gene expression in the thyroid [18]. As mentioned earlier, the hallmarks of ITD are markedly reduced or absent thyroidal uptake of radioiodide and reduced iodide S/P ratio. Absence of thyroidal iodide uptake is a typical feature of thyroid agenesis, and thus diagnosis is erroneously assigned to some patients with iodide trapping defect, especially when goiter was not present. Szinnai et al. [14] summarized the clinical features, laboratory findings, and mutations of the NIS gene in patients with ITD. Among these 31 patients, radioiodide uptake ranged from <1% to 4.8% and the iodide S/P ratio ranged from 0.94 to 5.2. Regarding goiter, 18 patients developed goiter and its diagnosis was made at a median age of 11 years. In our patient, the value of radioiodide uptake was higher than that in previously reported patients. However, the iodide S/P ratio in our patient was low. An iodide S/P ratio in the vicinity of 1 is considered to be the consequence of complete ITD while an iodide S/P ratio of up to 20 is considered to represent partial ITD [13]. Therefore, we initially speculated that the cause of CH in our patient was partial ITD due to defective NIS. However, sequence analysis showed that the NIS gene was normal; instead a mutation causing R31H in the PAX8 protein was found. As mentioned earlier, PAX8 gene expression during the fetal period is observed in the developing thyroid and kidney during human development [3]; however, its expression in salivary glands has not been examined. It is tempting to speculate that PAX8 is expressed and enhances NIS gene expression during embryogenesis in the human salivary glands similar to in the thyroid gland. Thus, PAX8 mutation may impair NIS function not only in the thyroid but also in the salivary glands. This possibility must be studied further.

In conclusion, we reported a patient with a mutation causing R31H substitution in the PAX8 protein, which manifested as partial ITD.

## Figures and Tables

**Table 1:** Laboratory findings in the patient.

Values of filter paper at neonatal screening	
TSH (mU/L) (normal range 0.1 ~ 10)	62.8
Values at the time of the first evaluation (Serum)	
17 days of age	
TSH (mU/L) (normal range 0.34 ~ 3.5)	202.7
T4 (nmol/L) (normal range 59.2 ~ 161.2)	91.2
T3 (nmol/L) (normal range 1.22 ~ 2.76)	1.88
^123^I thyroid scan (%)^a^ at 1, 3, 24 h	4.8, 5.8, 2.9
Saliva/Serum ^123^ratio^b^ at 2 h and 4 h	4.5, 3.8

Thyroid scan was performed at age of 6 years a, normal above 10%–35%, b, normal above 20.
